# An immunochromatographic strip sensor for marbofloxacin residues

**DOI:** 10.1371/journal.pone.0299709

**Published:** 2024-03-29

**Authors:** Xingdong Yang, Qingmei Li, Sharon Kwee, Jifei Yang, Qianqian Zhang, Xiaofei Hu

**Affiliations:** 1 Institute of Food and Drug Inspection, Zhoukou Normal University, Zhoukou, P. R. China; 2 Key Laboratory of Animal Immunology of the Ministry of Agriculture, Henan Provincial Key Laboratory of Animal Immunology, Henan Academy of Agricultural Sciences, Zhengzhou, P. R. China; 3 Department of Biomedical Engineering, University of Texas at San Antonio, San Antonio, TX, United States of America; Kerman University of Medical Sciences, ISLAMIC REPUBLIC OF IRAN

## Abstract

Marbofloxacin (MBF) was once widely used as a veterinary drug to control diseases in animals. MBF residues in animal food endanger human health. In the present study, an immunochromatographic strip assay (ICSA) utilizing a competitive principle was developed to rapidly detect MBF in beef samples. The 50% inhibitory concentration (IC_50_) and the limit of detection (LOD) of the ICSAs were 2.5 ng/mL and 0.5 ng/mL, respectively. The cross-reactivity (CR) of the MBF ICSAs to Ofloxacin (OFL), enrofloxacin (ENR), norfloxacin (NOR), and Ciprofloxacin (CIP) were 60.98%, 32.05%, 22.94%, and 23.58%, respectively. The CR for difloxacin (DIF) and sarafloxacin (SAR) was less than 0.1%. The recovery rates of MBF in spiked beef samples ranged from 82.0% to 90.4%. The intra-assay and interassay coefficients of variation (CVs) were below 10%. In addition, when the same authentic beef samples were detected in a side-by-side comparison between the ICSAs and HPLC‒MS, no statistically significant difference was observed. Therefore, the proposed ICSAs can be a useful tool for monitoring MBF residues in beef samples in a qualitative and quantitative manner.

## Introduction

Marbofloxacin (MBF) is a synthetic third-generation fluoroquinolone (FQ) antibiotic. Since the middle of the 1990s, MBF has been used exclusively in veterinary practice to treat respiratory, digestive, urinary, and skin infections in pets, such as dogs and cats, in Europe and the United States [1]. MBF blocks the growth of gram-negative pathogens, some gram-positive pathogens and Mycoplasma by inhibiting DNA transcriptase [2]. Additionally, MBF has been proposed for treating disease in the respiratory tract, soft tissues, digestive tract and breast tissue of food-producing animals (cattle, swine) since 1997 [3]. While MBF use was permitted in companion animals, many countries prohibited its use in all edible animals to reduce FQ-resistant campylobacter. MBF residues in animal tissues and milk can even cause the development of resistant strains of bacteria, allergic hypersensitivity reactions, etc., in the human body [4]. To prevent MBF residues from entering the food chain and reduce antibiotic resistance, many countries have also set maximum residue limits (MRLs) for MBF. The European Union (EU) has specified MRLs for MBF as follows: 150 μg/kg in muscle, liver and kidney, 50 μg/kg in fat and 75 μg/kg in milk (Commission Regulation of the EU No 37/2010). In China, MBF is not permitted for use in animals. However, because of its wide antimicrobial spectrum, MBF has been illegally used by animal producers to treat animal diseases [5]. The Chinese Ministry of Agriculture and Rural Affairs (Bulletin NO.89) approved the registration of three MBF injection drug products (CEVA SANTE ANIMALE S.A.) for veterinary use in China and has set MRLs in bovine adipose tissue to be 50 μg/kg for MBF. Therefore, the ability to monitor MBF residues in animal-derived foods is important.

Existing analytical methods to determine MBF in biological samples include high-performance liquid chromatography (HPLC) [6], HPLC-tandem mass spectrometry (HPLCLC‒MS/MS) [7, 8], reversed-phase HPLC (RP-HPLC) [9] and HPLC with ultraviolet detection [10] or fluorescence detection [11]. Although these methods exhibit relatively high sensitivity and good selectivity for detecting MBF, they involve several drawbacks, as chromatography is time-consuming, labour intensive, and limited to laboratory use due to its dependency on complex sample pretreatment, large and expensive instruments, and professional technicians. Furthermore, these drawbacks limit the amount of screening that can be feasibly performed. In contrast, immunoassays could overcome some of these shortcomings. While enzyme-linked immunosorbent assays (ELISAs) eliminate the need for sample pretreatment and certain matrix interferences can be eliminated by washing between steps, ELISAs require a laboratory setting, technical training, and multiple steps. Compared with colloidal gold-labeled immunochromatography strip assays (ICSAs), the novel immunosensors need to be further validated for practical application in the market, although they have the advantages of low manufacturing cost, simple storage conditions, no/minimal sample preparation, and immediate operability [12, 13]. ICSAs offer a simpler and more convenient solution, and previous studies demonstrated that this method is effective in detecting fluoroquinolones immunosorbent [14–25]. ICSAs’ principal advantage over other analytical methods is that it does not require specialized skills, expensive and complex instrumentation, all-inclusive, can provide results in a timely manner (5–10 min), and can easily yield both qualitative and quantitative results for a wide range of analytes.

**Fig 1:**
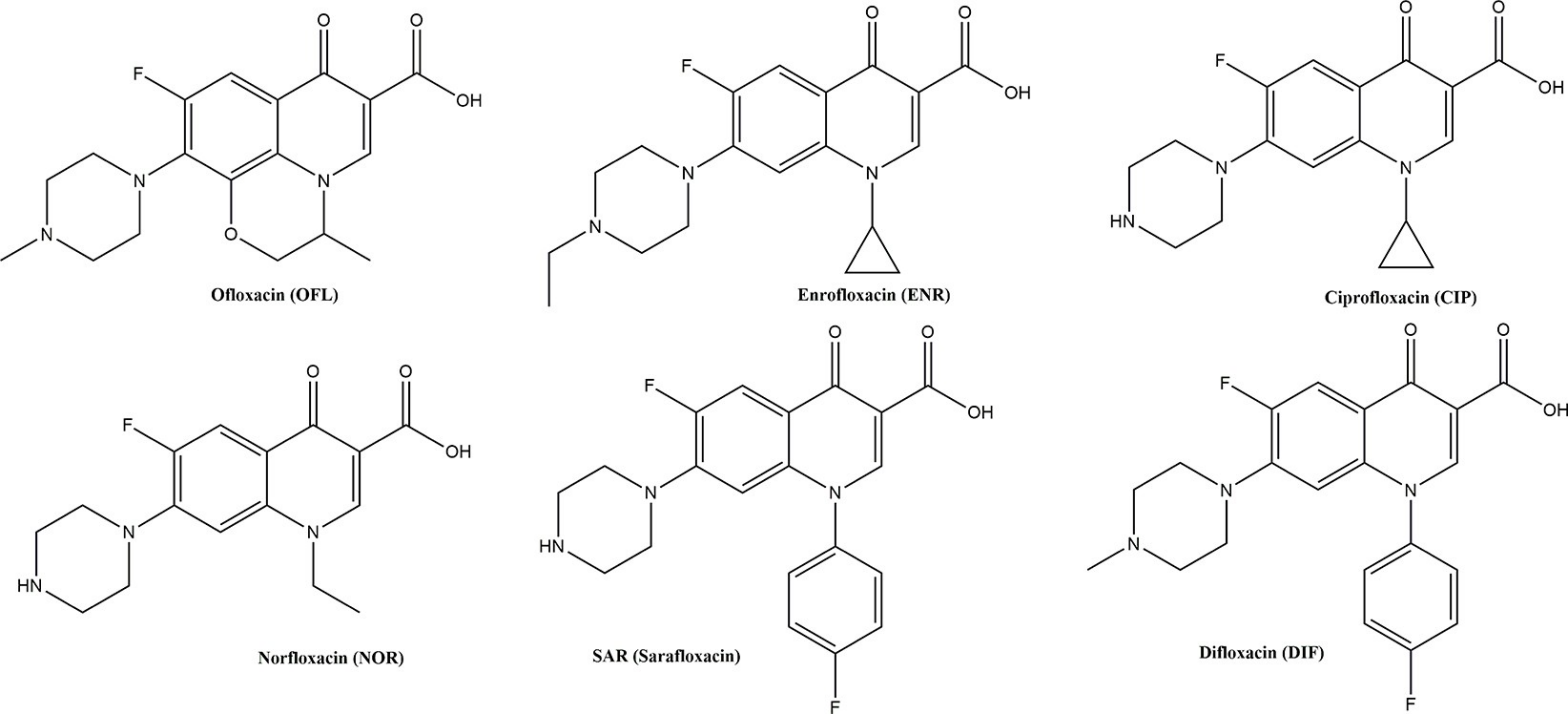
Chemical structure of FQs for mAbs produced in previous literature.

Monoclonal antibodies (mAbs) of FQ mainly focused on SAR (Sarafloxacin), Enrofloxacin (ENR), Ciprofloxacin (CIP), Norfloxacin (NOR), Ofloxacin (OFL) and Difloxacin (DIF) in ELISAs [26] and ICSAs [27, 28] based on those mAbs had been developed to detect FQ in animal feeds, livestock carcasses and milk samples (Fig 1). Although MBF is widely used in veterinary clinics, the need for a method to simply detect MBF residues in food has rarely been addressed [4, 29].

The objective of this study was to develop an ICSA for the rapid detection of MBF in beef samples. MBF was coupled with bovine serum albumin (BSA) and ovalbumin (OVA) via a mixed EDC/NHS method. The mAbs against MBF were produced and then conjugated with colloidal gold nanoparticles (CGNs) for the probe. The ICSA utilizes a competitive principle and was validated in terms of specificity, sensitivity, accuracy, and precision in spiked recovery experiments and verified by HPLC‒MS for use in detecting authentic edible animal tissue samples.

## Materials and methods

### Chemicals and materials

MBF, N-hydroxy succinimide (NHS), N, N-dimethylformamide (DMF), Na_2_B_4_O_7_, 1-(3-(dimethylamino) propyl)-3-ethylcarbodiimide hydrochloride (EDC), Freund’s adjuvant, and a mouse monoclonal antibody isotyping kit were purchased from Sigma (St Louis, MO, USA). ENR, CIP, NOR, OFL, and DIF were purchased from Macklin Biochemical Co., Ltd. (Shanghai, China). HRP-conjugated goat-anti-mouse IgG was purchased from Sino-American Biotechnology Co., Ltd. (Luoyang, China). BSA and OVA were both obtained from Yuanye Biotechnology Co., Ltd. (Shanghai, China). Filter membranes, nitrocellulose (NC) membranes, absorbent pads, and glass fibers were purchased from Millipore (Bedford, MA, USA).

### Preparation of immunogen, coating antigen and anti-MBF mAbs

The EDC/NHS method for preparing the MBF-BSA and MBF-OVA conjugates was modified from a previous report [30, 31]. Briefly, 18.12 mg MBF was dissolved in 3 mL DMF and mixed with 5.75 mg NHS and 9.59 mg EDC at room temperature (RT) for 12 h. The mixture was centrifuged at 3950 g for 5 min to collect the supernatant. In an ice bath, the active final products were slowly added dropwise into 2 mL phosphate buffer saline (PBS, 0.01 M) containing 33.22 mg of BSA or 22.25 mg OVA, and the mixture was stirred for 12 h at 4°C. The final mixture was dialyzed against PBS nine times for three days and centrifuged at 3000 g for 15 min at 4°C. The supernatant was collected and stored at -20°C.

Experiments were performed after obtaining approval from the Animal Ethics Committee of Zhoukou Normal University (ZKNU2021038). All methods were performed in accordance with the relevant guidelines and regulations. Three BALB/c mice (7 weeks of age) were subcutaneously immunized with 70 *μ*g immunogen MBF-BSA at intervals of 3 weeks five times. Freund’s complete adjuvant and Freund’s incomplete adjuvant with isometric immunogen were used for the first and 2–5 immunizations, respectively. The titer and specificity of antiserum in mice were detected by indirect and indirect competitive ELISAs (ic-ELISAs) using microplate readers (Multiskan FC, Thermo Fisher Scientific Instrument Co., Ltd., USA) as previously described [32, 33]. The mouse that provided the best half-maximal inhibitory concentration (IC_50_) in the ic-ELISA was selected to be vaccinated intraperitoneally with 100 *μ*g of immunogen before cell fusion. Each animal’s behaviors and appearance were inspected daily according to ARRIVE guidelines for humane endpoints before the end of the study. Accordingly, if mice appeared distressed, failed to eat, or lost 20% of their body weight within a week, they were euthanized. Body weights and granuloma burden were recorded weekly. Twenty-four-hour food intake was measured during week 12 of the experimental study to ensure that food intake was not compromised by the granuloma burden. At the end of the protocol at week 16, mice were euthanized with isoflurane anesthesia for three minutes, followed by cervical dislocation. Then splenectomy was performed.

The detailed cell fusion between splenocytes and myeloma cells procedure was described in a previous study [34, 35]. Briefly, hybridomas secreting anti-MBF mAbs were selected by ic-ELISA, subcloned with limiting dilution, and cultured to prepare ascites fluids in paraffin-primed mice. The isotype of the mAb was determined using a mouse mAb isotyping kit. The measurement of mAb affinity constant (Ka) was carried out according to the procedure described by Batty et al [36]. Ka can be calculated according to the following formula.


Ka=(n−1)/2(n[Ab']t−[Ab]t)

n=[Ag]t/[Ag']t

[Ag] *t* and [Ag’] *t* represents two different concentrations of coating antigen, and [Ab] *t* and [Ab’] *t* represents the corresponding concentrations of the mAb.

### Conjugation of anti-MBF mAbs with colloidal gold and preparation of ICSAs

Anti-MBF mAb 7A12 was labeled with spherical colloidal gold nanospheres (CGNs). The CGNs were produced by deacidizing HAuCl_4_ with sodium citrate reduction [37]. To conjugate the anti-MBF mAb 7A12 with CGNs, the antibody concentration and pH value were adjusted to the best conditions with 10% NaCl and 0.2 M K_2_CO_3_, respectively. To produce CGN-mAbs, 1.5 ml mAb 7A12 solution (2 μg/mL) was incubated with 7.5 mL colloidal gold solution (pH 9.0) for 25 min at RT. The mixture was incubated at RT for another 12 min with 1.5 mL of 10% BSA. The labeled mAbs were then washed twice and centrifuged at 15000 × g for 30 min at 4°C. The sediment was finally resuspended in sodium borate solution containing 1% BSA (W/V) and stored at 4°C before use. To capture hapten MBF or MBF-BSA, CGN-mAbs were sprayed on the conjugate pad. ICSAs were assembled according to a previously described method [38, 39].

### Sample pretreatment for the ICSAs

Fresh beef was purchased from a local market. It was determined by HPLC‒MS to be negative for MBF. The beef sample was then minced and homogenized. The tissue sample (2±0.01 g) was transferred into 50-mL centrifuge tubes and dissolved in PBS (8 mL) at a ratio of 1:4 to prepare a 0.25 g/mL negative beef solution. MBF was spiked with negative beef solution to give final concentrations of 6, 10, 20, 80, 160, and 320 ng/mL. The sample was then extracted by adding 5 mL of 5% trichloroacetic acid and acetonitrile solution containing 1% acetic acid (8:2, v/v) and vibrating for 10 min. Following centrifugation at 10000 × g for 10 min at 4°C, 1.5 mL of the supernatant and 2 mL of n-hexane were added successively in a 10-mL centrifuge tube and vortexed for 1 min. After 5 min, 1 mL of the subnatant was filtered using a 0.22 *μ*m microporous membrane.

### Evaluating the performance of ICSAs

The sensitivity of the ICSAs was determined using standard solutions serially diluted with different concentrations of MBF (0, 1.0, 2.0, 4.0, 8.0, 16.0 and 32.0 ng/mL). After approximately 150 μL of sample was dropped onto the sample pad, qualitative detection results were evaluated by the appearance of colouration at the T and C lines within 8 min. For quantitative detection, a TSR3000 membrane strip reader (Bio-Rad, USA) was used to read the relative optical density (ROD) of the T line. The linearity is between B/B_0_ and the log of the MBF concentration. B and B_0_represent the ROD values of the testing samples and the blank sample, respectively. Using GraphPad Prism, the IC_50_ value was computed based on the linear regression equation.

To evaluate the specificity of the ICSAs, MBF and its structural analogues (OFL, ENR, NOR, CIP, DIF, and SAR) in the negative beef samples were tested. Cross-reactivity (CR) was assessed with the equation CR (%) = (IC_50_ of MBF)/IC_50_ of the competitor) × 100.

The accuracy and precision of the ICSAs were evaluated by detecting beef samples containing 5.0, 30.0, and 100.0 ng/ml MBF six times. The accuracy and precision were expressed as recovery and coefficient of variation (CV, %), respectively.

In a previous study, anti-MBF polyclonal antibody was prepared and applied in ic-ELISAs for the determination of MBF in Beef and Pork [4]. However, The IC_50_ of the ELISA for MBF in real muscle extracts was far higher than 22.0 ng/mL, which is much higher than ours. Ic-ELISAs revealed that the mAb against MBF (M4E3) exhibited the highest sensitivity with an IC_50_ of 0.07 ng/mL and a LOD of 0.01 ng/mL for detection of MBF. The recovery rate of MBF in milk ranged from 72.28% to 129.19%. Furthermore, a visual CGNs-based immunochromatographic assay was developed for detecting MBF with a cut-off value of 1 ng/mL in both PBS and a milk sample by using this mAb [29]. However, the immunochromatographic assay only performed qualitative detection of MBF residues in milk, not quantitative detection.

### Authenticity of ICSAs

The negative tissue samples spiked with four different MBF concentrations (0, 8.4, 25.8, and 67.6 ng/mL) were detected in parallel by ICSAs and HPLC‒MS. HPLC‒MS was performed for instrumental analysis of MBF using a previous method with minor changes [7]. Briefly, liquid chromatography conditions were as follows: Diamonsil C18 (2.0×150 mm×5 μm), the mobile phases were 1000 mL of double distilled water, 1 mL of formic acid and 500 μL of 1 M ammonium formate solution, the flow rate was 0.25 mL/min, column temperature was 30°C, and the MBF was monitored at 295 nm. The mass spectrometry conditions were as follows: electrospray ion source, positive ion scanning, auxiliary gas (N_2_) at50 psi and 350°C, flow rate of auxiliary gas: 9.0 L/min, capillary outlet voltage of 115 V, and collision energy of 12 eV. No peak was observed at 295 nm for beef samples spiked without MBF (0 ng/mL). MS also provided negative results.

## Results and discussion

### Preparation of complete antigen

**Fig 2:**
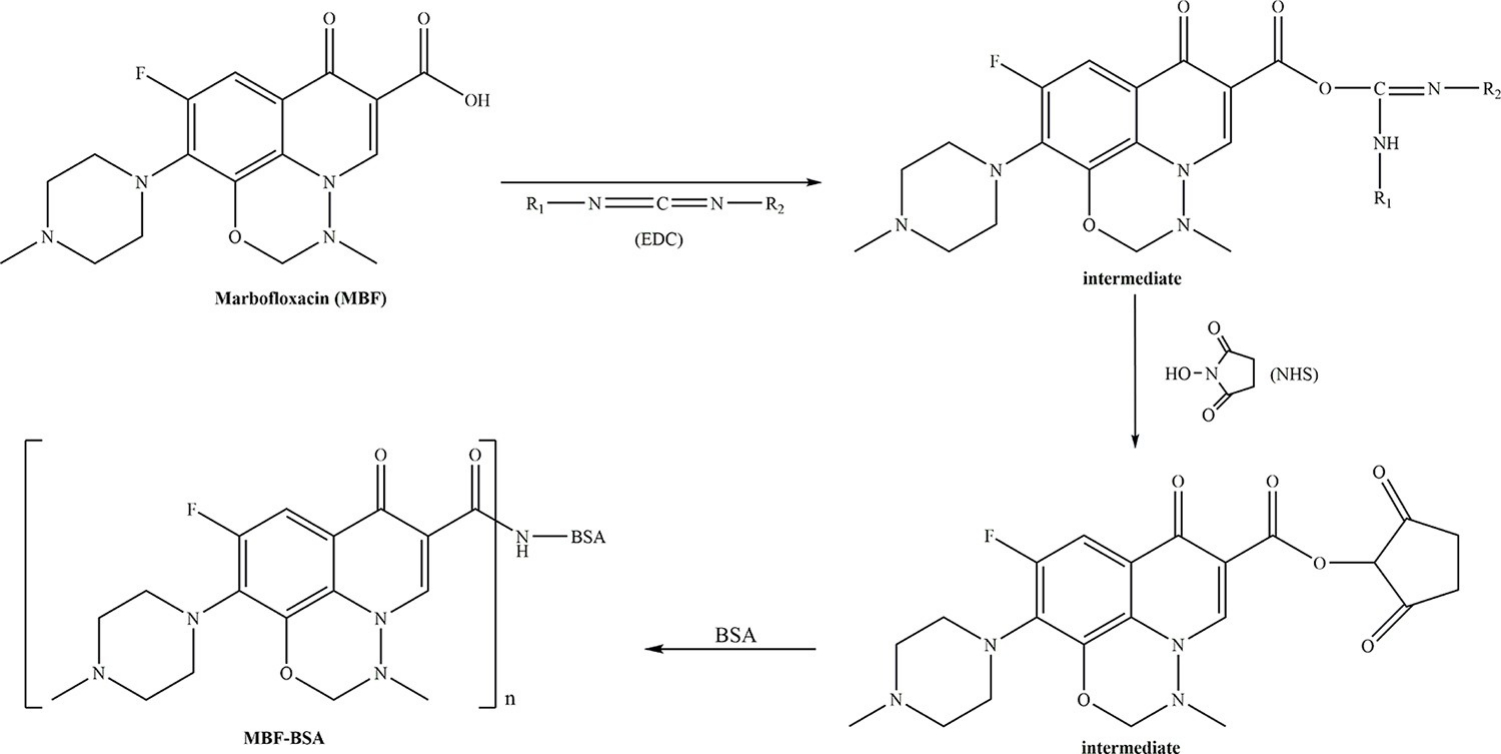
Synthesis of MBF-BSA using an activated ester method.

The molecular weight of hapten MBF is 362.35Da. Hapten MBF can bind to homologous antibodies, exhibits no immunogenicity, and does not stimulate the immune response of humans and animals. Complete antigen MBF-BSA is necessary to produce specific antibodies against MBF. Haptens with carboxyl groups are normally coupled with carrier proteins using one of the following methods: the EDC/NHS method or the mixed anhydride (MA) method. The conjugating conditions in the EDC/NHS method need to be completely anhydrous, but compared with the MA method, this method produces more outgrowth. Therefore, we synthesized the immunogen MBF-BSA and coating antigen MBF-OVA using the EDC/NHS method (Fig 2). The coupling rates of MBF with BSA and OVA were calculated by ultraviolet (UV) scanning at 19.2:1 and 14.6:1, respectively. An immunized mouse with an antibody titer of 1:5.12×10^4^ (S1 Table) and an IC_50_ value of 20.5ng/mL (S2 Table and S1 Fig) was chosen for subsequent cell fusion.

### Characterization of mAbs against MBF

The 3F4, 3F10, 7A12, and 8A4 cell lines were obtained after screening. mAbs 7A12, which showed the highest antibody titer of 1:1.024×10^6^ (S3 Table) and the lowest IC_50_ value of 2.29 ng/mL (S4 Table and S2 Fig), was selected to be labeled by CGNs with a diameter of 25 nm. The Ka and the subtype of 7A12 were determined by ELISA to be 1.2×10^10^ L/mol and immunoglobulin G 1, respectively.

In previous studies of immunoassays for the detection of MBF residues, the IC_50_ of ic-ELISA using mAbs developed by Sheng et al. was 4.6 ng/mL in phosphate buffer, which is much higher than ours, suggesting that the ic-ELISA in this study is more sensitive.

### Test procedure and principle of ICSAs

**Fig 3:**
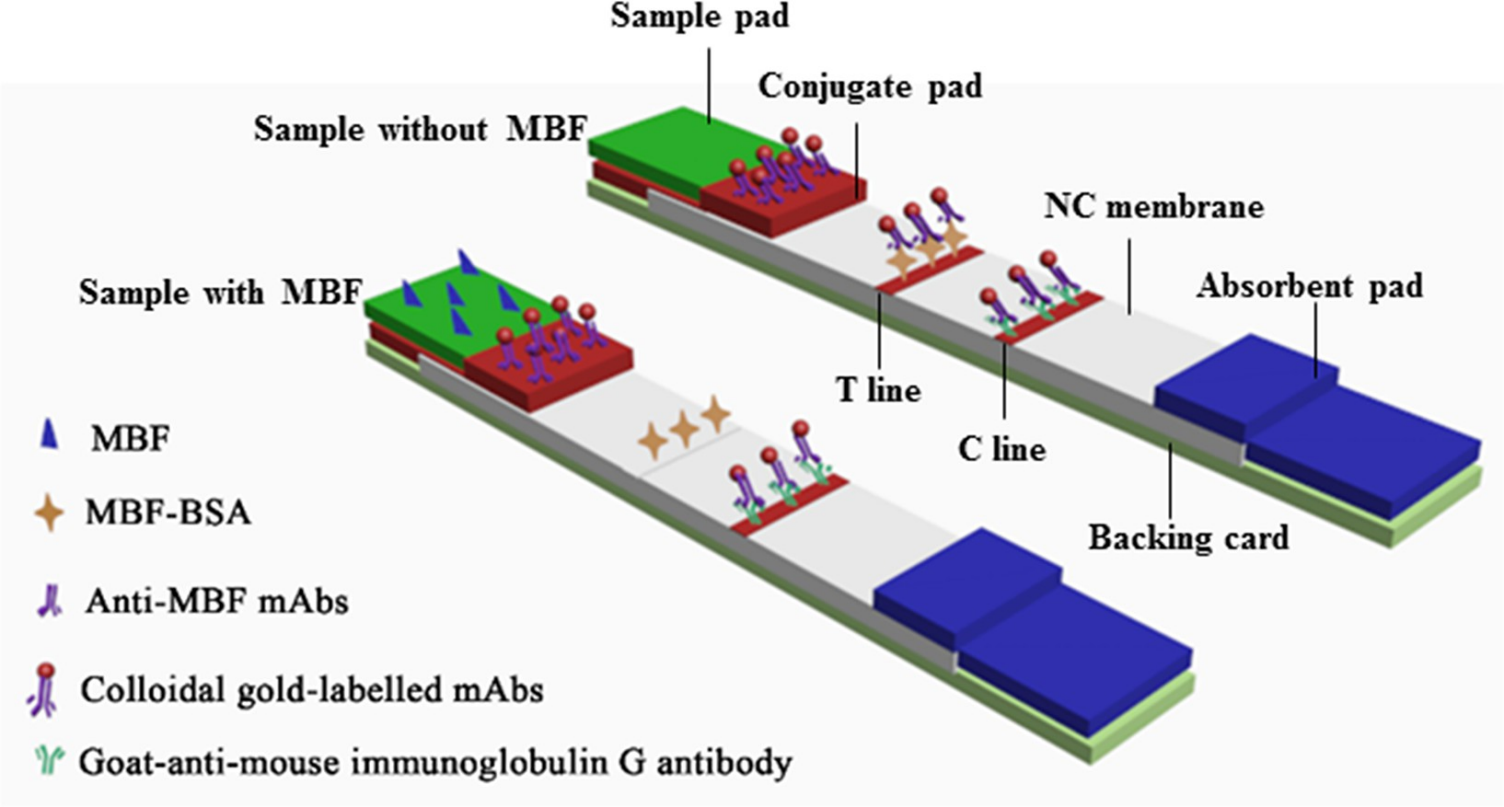
Structure and schematic diagram of the ICSAs.

The ICSA is composed of a sample pad, a conjugate pad, an NC membrane, an absorbent pad, and a backing card. The operating principles of the ICSAs involved using free MBF in the sample solution to compete with MBF-BSA for binding to CGN-mAbs at the test line (T line). The sample solution is added to the sample pad and flows towards the absorbent pad via the capillary effect. If MBF is present in the sample, it will bind to the CGN-mAbs, decreasing the amount of CGN-mAbs available to bind with MBF-BSA at the T line; the CGN-mAbs will then be captured by a second antibody (goat-anti-mouse IgG) at the control line (C line). Thus, the T line will show a light red or achromatic color that is inversely proportional to the amount of MBF present in the sample, while the C line is shown as red. Within the linear range, the concentration of MBF in the sample solution is inversely correlated with the intensity of red at the T line. If no MBF is present in the tested sample, the CGN-mAbs will bind to the MBF-BSA at the T line and the second antibody at the C line. Note that for a valid ICSA, a visible red of the C line should always appear. Otherwise, the test strip is invalid, and a new test strip should be used (Fig 3) [40].

### Sensitivity of the ICSAs

**Fig 4:**
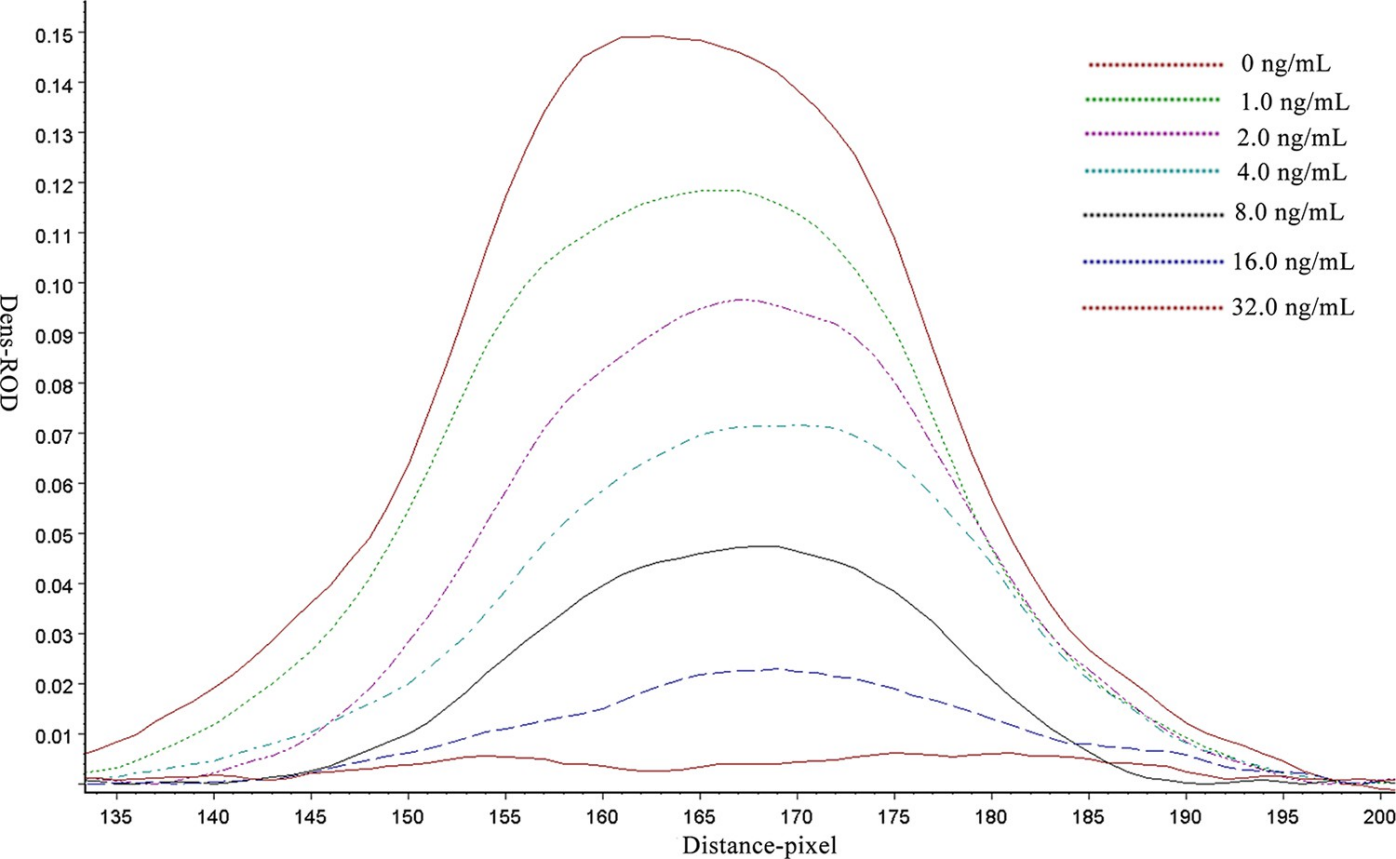
ROD curves of beef samples containing MBF at 0, 1.0, 2.0, 4.0, 8.0, 16.0 and 32.0 ng/mL were detected by ICSAs using a strip reader. The color of the test line is depicted in the figure.

**Fig 5:**
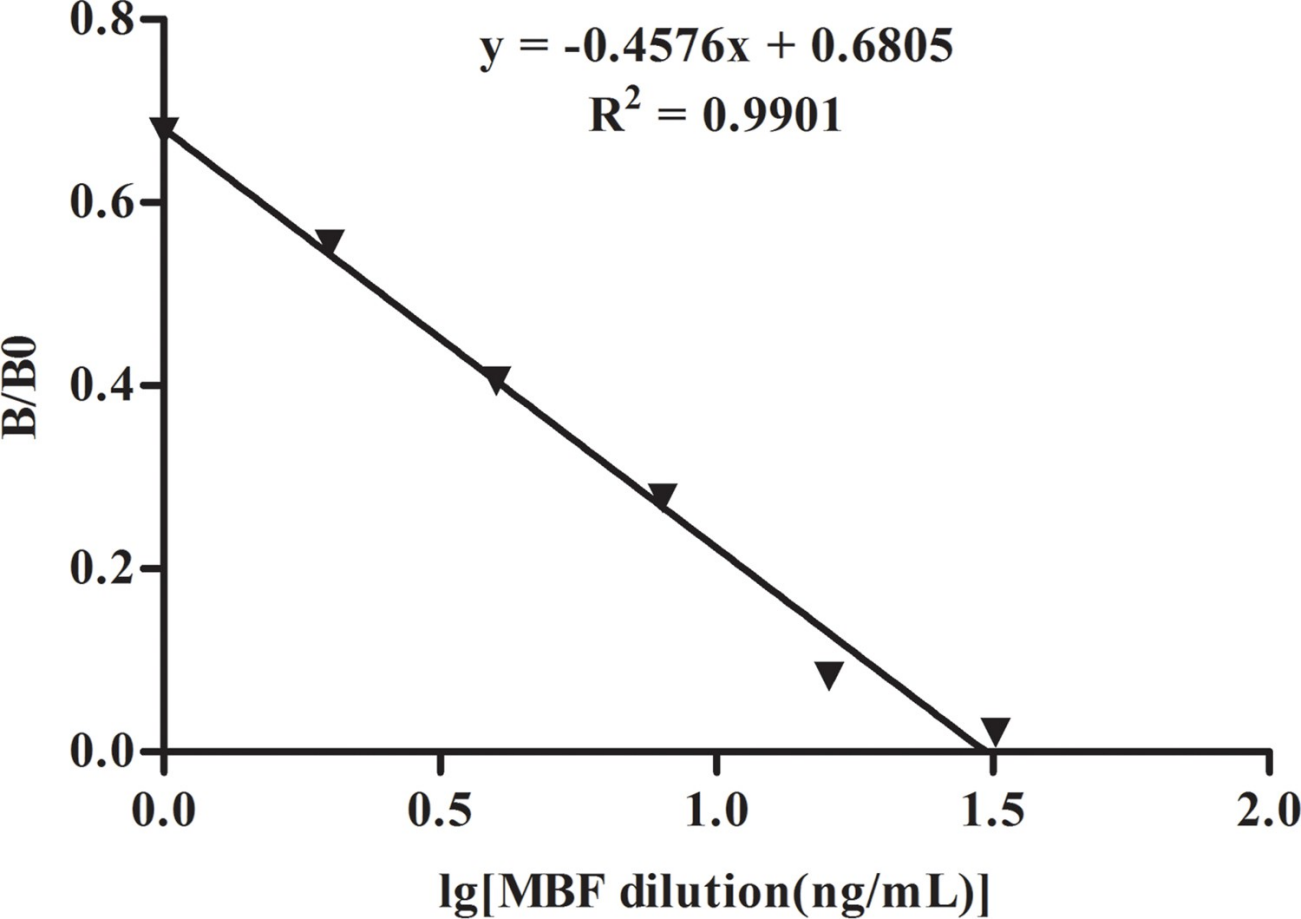
The quantitative calibration curve for MBF using ICSAs. The X-axis shows the logarithmic concentrations of MBF. B/B_0_ represents the percentage of ROD values determined by the different concentrations of MBF in beef samples divided by the zero-dose.

To demonstrate the sensitivity of ICSAs for MBF, we detected reference solutions of MBF at concentrations of 0, 1.0, 2.0, 4.0, 8.0, 16.0 and 32.0 ng/mL by the ICSAs. The ROD value for each ICSA was read by scanning the T line with a TSR3000 membrane strip reader (Fig 4 and Table 1). The linearity is between B/B_0_ and the log of MBF concentration in beef samples (Fig 5). ROD values were negatively correlated with the MBF concentration samples. The regression equation was y = -0.4576 + 0.6805 (R^2^ = 0.9901). According to the linear equation, the IC_50_ value was 2.5 ng/mL, and the limit of detection (LOD) was 0.5 ng/mL. The ranges are defined as extending from the IC_20_ to the IC_80_. The detection ranges of the ICSA were 0.5–11.2 ng/mL for MBF. The qualitative LOD of the ICSAs was identified to be 4.0 ng/mL based on unaided visual assessment.

**Table 1:** ROD of ICSAs using spiked beef samples.

MBF concentration (ng/mL)	G/D×area-ROD (pixel)	G/Peak-ROD (pixel)
0	280.959	0.149
1.0	190.120	0.119
2.0	155.660	0.097
4.0	113.842	0.072
8.0	77.891	0.047
16.0	23.107	0.023
32.0	5.914	0.006

### Specificity of the ICSAs

The specificity of the ICSAs was evaluated in comparison with other structural analogues of MBF, including OFL, ENR, NOR, CIP, DIF, and SAR. When these competitors were added at a final concentration of 1000 ng/ml, the colour of the T lines remained negative. The CR of the ICSAs based on mAb 7A12 to OFL, ENR, NOR, and CIP were 60.98%, 32.05%, 22.94%, and 23.58%, respectively; however, the CR for DIF and SAR was less than 0.1% (Table 2 and S5 Table). One explanation is that FQs, such as OFL, ENR, NOR, CIP, DIF, SAR, and MBF, possess the same or similar basic chemical structures (for example, a fluorine atom in the C-6 position of the quinoline ring and piperazinyl at C-7), but DIF and SAR have an additional unique fluorobenzene structure. The MRLs established by the EU for FQs in muscle, kidney, and egg were greater than 30 ng/mL. Therefore, the ICSAs of MBF are passable tools to test OFL, ENR, NOR, and CIP residues in animal food.

**Table 2:** CR of ICSAs with competitors.

Compounds	IC_50_ (ng/mL)	CR (%)
Marbofloxacin (MBF)	2.5	100
Ofloxacin (OFL)	4.1	60.98
Enrofloxacin (ENR)	7.8	32.05
Norfloxacin (NOR)	10.9	22.94
Ciprofloxacin (CIP)	10.6	23.58
Difloxacin (DIF)	> 300	< 0.1
Sarafloxacin (SAR)	> 300	< 0.1

### Accuracy and precision of the ICSAs

Beef samples with MBF (5.0, 30.0, and 100.0 ng/mL) were tested. The ROD of the T line was scanned by the strip reader, and MBF concentrations were evaluated by a standard curve. The recoveries ranged from 82.0% to 90.4% (Table 3 and S6 Table). The intra-assay and interassay CVs of the ICSAs were less than 10.0%.

**Table 3:** Accuracy and precision of the ICSAs.

Spiked MBF (ng/mL)	Intra-assay			Inter-assay		
	Mean ± SD (ng/mL)	Recovery (%)	CV (%)	Mean ± SD (ng/mL)	Recovery (%)	CV (%)
3.0	2.46±0.19	82.0±6.3	7.58	2.39±0.23	79.7±7.7	9.63
5.0	4.15±0.30	83.1±6.0	7.19	4.27±0.39	85.4±7.8	9.13
15.0	13.16±0.76	87.7±5.1	5.78	12.86±0.77	85.7±5.1	5.94
30.0	26.15±1.45	87.2±4.8	5.53	25.99±1.57	86.6±5.2	6.03
100.0	90.35±3.37	90.4±3.4	3.73	91.03±3.82	91.0±3.8	4.20

### Comparison of ICSAs with HPLC‒MS

ICSAs and HPLC‒MS were performed to test beef samples. Statistical analysis using a t-test showed no significant difference between the results using the two methods (Table 4 and S7 Table); thus, the ICSAs are reliable for the range of values tested.

**Table 4:** Comparison between the ICSAs and HPLC-MS.

MBF (ng/mL) in beef samples	ICSAs (ng/mL)	HPLC (ng/mL)
8.4	7.06±0.74^***a***^	7.32±0.51^***a***^
25.8	22.20±1.25^***a***^	24.12±0.68^***a***^
67.6	59.60±2.48^***a***^	64.29±1.28^***a***^

^***a***^ the superscript represents no statistical significance between the results given by the ICSAs and HPLC (P > 0.05)

## Conclusion

In this study, based on obtaining mAb against MBF, ICSAs were established for detecting MBF residues in beef samples. The immunosensor is characterized by high sensitivity, specificity, accuracy and precision. The developed test system for MBF was shown to be efficient for the detection of this antibiotic in food matrices and can be considered an efficient on-screening tool for the rapid food quality and safety control.

Although ICSA has the advantages of low price, easy operation, rapid detection, sensitivity, specificity and accuracy, it also has some disadvantages, such as qualitative testing, low signal strength and poor quantitative discrimination. However, most of the tests require quantitative testing, and with further research, semiquantitative and quantitative testing of ICSAs is gradually being refined. Further research is needed on multiplex ICSA technology for the simultaneous detection of multiple samples and on ultrasensitive quantitative and accurate detection techniques.

## Supporting information

S1 Checklist *PLOS ONE* humane endpoints checklist. (DOCX)

S1 Table Determination of teters of mice polyantiserum against MBF. (XLSX)

S2 Table Determination of sensitivity of mice polyantiserum against MBF. (XLSX)

S3 Table The indirect ELISA titer of mAbs against MBF. (XLSX)

S4 Table Inhibitive titers of mAbs against MBF. (XLSX)

S5 Table Relative optical density of ICSAs against fluoroquinolones. (XLSX)

S6 Table Recovery of the ICSAs for MBF spiked in beef samples. (XLSX)

S7 Table Comparison of the ICSAs with HPLC for three levels of MBF residues in beef samples. (XLSX)

S1 Fig Inhibition curve of polyantiserum from immunised mice. (A) Mouse #1. (B) Mouse #2. (C) Mouse #3.
(TIF)

S2 Fig Inhibition curves of mAbs against MBF. (A) mAb 3F4. (B) mAb 3F10. (C) mAb 7A12. (D) mAb 8A4.
(TIF)
