# Supplementary figures and images for: An immunochromatographic strip sensor for marbofloxacin residues
Source: PLoS One. 2024 Mar 29;19(3):e0299709. doi: 10.1371/journal.pone.0299709 (PMC10980191; doi:10.1371/journal.pone.0299709)

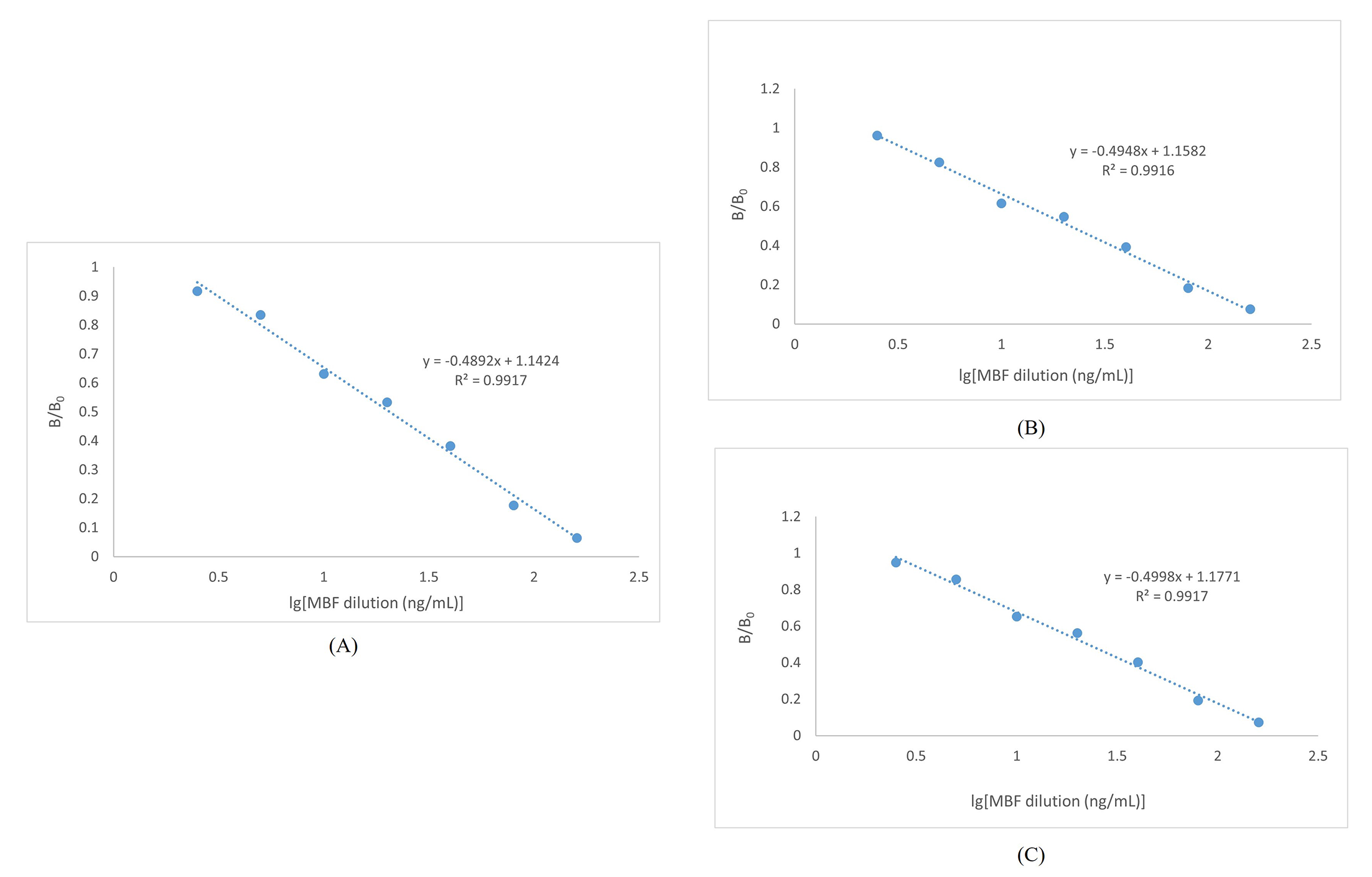

Supplement: S1 Fig — (A) Mouse #1. (B) Mouse #2. (C) Mouse #3. (TIF) [file pone.0299709.s009.tif]

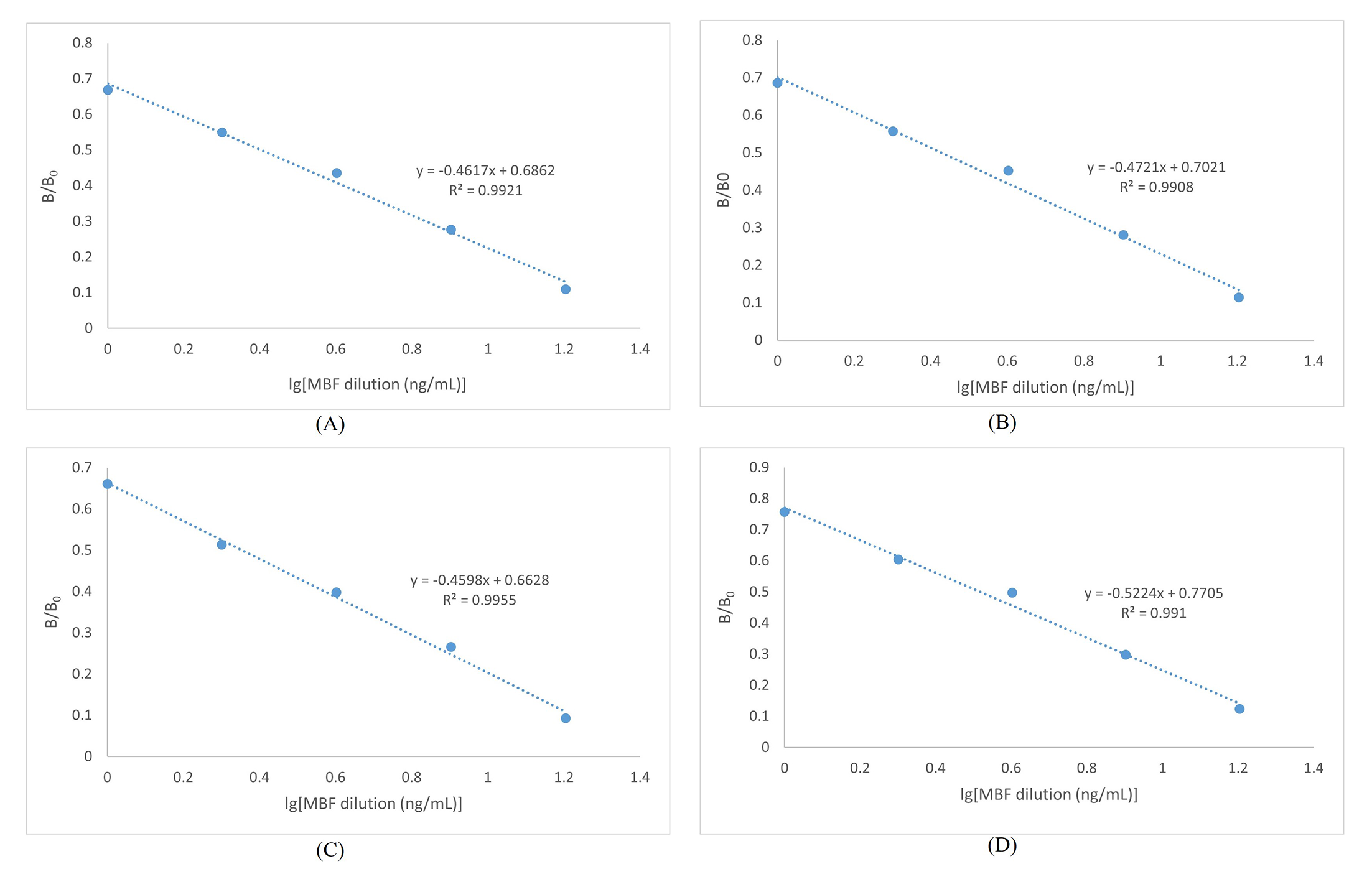

Supplement: S2 Fig — (A) mAb 3F4. (B) mAb 3F10. (C) mAb 7A12. (D) mAb 8A4. (TIF) [file pone.0299709.s010.tif]
